# Independent and Combined Effects of Antioxidant Supplementation and Circuit Resistance Training on Selected Adipokines in Postmenopausal Women

**DOI:** 10.3389/fphys.2019.00484

**Published:** 2019-04-26

**Authors:** Ayoub Saeidi, Georges Jabbour, Mehdi Ahmadian, Asieh Abbassi-Daloii, Fatemeh Malekian, Anthony C. Hackney, Saber Saedmocheshi, Gholam Basati, Abderraouf Ben Abderrahman, Hassane Zouhal

**Affiliations:** ^1^Exercise Biochemistry Division, Faculty of Physical Education and Sports Sciences, University of Mazandaran, Babolsar, Iran; ^2^Sport Science Program, College of Arts and Sciences, Qatar University, Doha, Qatar; ^3^Department of Physical Education and Sports Sciences, Islamic Azad University, Aliabad-e Katul, Iran; ^4^Department of Sports Physiology, Islamic Azad University, Amol, Iran; ^5^Southern University Agricultural Land Grant Campus, Baton Rouge, LA, United States; ^6^Department of Exercise & Sports Science, The University of North Carolina at Chapel Hill, Chapel Hill, NC, United States; ^7^Exercise Physiology Division, Faculty of Sport Science, Birjand University, Birjand, Iran; ^8^Department of Clinical Biochemistry, Faculty of Medicine, Ilam University of Medical Sciences, Ilam, Iran; ^9^Higher Institute of Sport and Physical Education of Ksar Saïd, Tunis, Tunisia; ^10^Movement Sports Science Laboratory, University of Rennes, Rennes, France

**Keywords:** postmenopausal women, Zataria Multiflora, adipokine, visfatin, vaspin, leptin, inflammation, metabolic syndrome

## Abstract

We examined the effects of the independent and combined effects of Zataria Multiflora supplementation and circuit resistance training (CRT) on selected adipokines among postmenopausal women. Forty-eight postmenopausal women were divided into four groups: Exercise (EG, *n* = 12), Zataria Multiflora (ZMG, *n* = 12), exercise and Zataria Multiflora (ZMEG, *n* = 12), and control (CG, *n* = 12). Participants in experimental groups either performed CRT (3 sessions per week with intensity at 55% of one-repetition maximum) or supplemented with Zataria Multiflora (500 mg every day after breakfast with 100 ml of water), or their combination, for 8 weeks. Blood samples were collected at pre- and post-intervention for measuring selected adipokines, including visfatin, omentin-1, vaspin, FGF-21, adiponectin, leptin, and ghrelin. Our findings demonstrated that visfatin, vaspin, and leptin levels significantly decreased over the intervention period (all *p* < 0.05), with these values were lower in EG and ZMEG in comparison to CG at post-intervention (all *p* < 0.05). Visfatin and vaspin levels were also lower in ZMEG in comparison to EG at post-intervention (both *p* < 0.05). In contrast, omentin-1, ghrelin, adiponectin, and FGF21 significantly increased in EG and EMG (all *p* < 0.05) after CRT. These findings suggest that Zataria Multiflora supplementation by itself has little effect on measured adipokines; however, its combination with CRT produced noticeable effects on circulating levels of these adipokines, even more than CRT alone. Consequently, a combination of CRT and Zataria Multiflora supplementation may represent a potentially beneficial non-pharmacologic intervention on some selected adipokines in postmenopausal women.

## Introduction

Menopausal transition is associated with weight gain, possibly by an increase in visceral and total body fat that usually originates form reduction in total energy expenditure as a consequence of physical inactivity, coupled with depression, age-induced sarcopenia, and lower basal metabolic rate (Davis et al., 2012; Al-Safi and Polotsky, 2015). It has also been reported that menopausal transition induces variations in the circulating levels of adipokines; however, it is unclear as to whether those changes could be explained by obesity (Sowers et al., 2008). Therefore, identifying and mapping strategies (i.e., exercise and healthy diet) to prevent and/or minimize gaining weight in postmenopausal individuals is of profound importance.

Adipokines regulates numerous biological processes in systemic organs, such as brain, liver, skeletal muscle, heart, and endocrine glands (Lehr et al., 2012). Deregulated production or secretion of these adipokines is reported with obesity, which could subsequently contribute in appetite and satiety disturbances, adipose tissue distribution, insulin sensitivity and secretion abnormalities, endothelial function, angiogenesis, inflammation, blood pressure, hemostasis, and osteoarticular functions and reproduction dysfunctions (Blüher, 2014). This deregulation of adipokines is exemplified by the direct and positive association between insulin resistance, diabetes, and obesity-related diseases with some adipokines, like leptin, vaspin, and visfatin, and an inverse association between those diseases and other adipokines, like omentin-1, adiponectin, and FGF-21 (Blüher, 2014; Fève et al., 2016). Consequently, any modulation, by exercise, supplementation, or their combination, on these substances may provide favorable prospects of mitigating obesity-related morbidities.

Amongst various forms of resistance training, circuit resistance training (CRT) is reported to improve maximum oxygen consumption, functional capacity, and body composition and strength (Camargo et al., 2008; Bocalini et al., 2012). While CRT is suggested as a time-efficient training modality that can elicit health benefits in various healthy and clinical populations, including postmenopausal individuals (Brentano et al., 2008; Williams et al., 2013), most clinical investigations have utilized aerobic exercise training (i.e., treadmill and cycling) as a strategy to induce desired changes in body composition and metabolic syndrome markers (Pearsall et al., 2014; Wiklund et al., 2014; Guadalupe-Grau et al., 2018; Mora-Rodriguez et al., 2018). It is reported, for example, that aerobic exercise training, alone or combined with hypocaloric diet, improve symptoms of the metabolic syndromes, presumably through changing the levels of inflammatory adipokines (You and Nicklas, 2008). Further, a newly released research revealed that 6 months of aerobic interval training improve the capacity for fat oxidation during exercise and enhance the maximal oxygen consumption, coupled with skeletal muscle improvement in mitochondrial enzyme activity (Guadalupe-Grau et al., 2018). Taken together, the effects of CRT on health-related parameters, such as body composition and metabolic syndrome markers, adipokines in particular, within clinical populations, especially postmenopausal, remain unanswered and needs to be investigated.

Herbal remedies, also known as herbal medicine, has for decades gained a great deal of interest among researchers all over the globe as a healthier alternative to medication in order to either prevent, alleviate, or minimize diseases outcomes (Ekor, 2014). Zatraia Multiflora (Aavishan-Shirazi) is one of those herbs which is popular for its positive impacts (e.g., antibacterial, antifungal, analgesic, and antioxidant- and immune-regulatory effects) on body function (Boskabady and Gholami Mhtaj, 2014). A recent study, in animal, found increased levels of adipokines following Zataria Multiflora supplementation (Mohammadi et al., 2014). These authors suggested the increased gamma peroxisome proliferator-activated receptor (PPAR) protein as a potential mechanism for this response. It is, therefore, plausible that Zataria Multiflora may have some effects on metabolic syndrome markers including adipokines. This underlines the needs for human-based research investigating the potential modulatory effects of Zataria Multiflora on adipokines, especially in postmenopausal individuals.

To the best of our knowledge, no study to date has examined the independent and/or combined effects of Zataria Multiflora supplementation either on selected adipokines or in postmenopausal individuals. Thus, the present study was conducted to provide some preliminary results on the effects of Zataria Multiflora supplementation and exercise training on some selected adipokines within postmenopausal.

## Materials and Methods

### Experimental Design and Study Population

**FIGURE 1:**
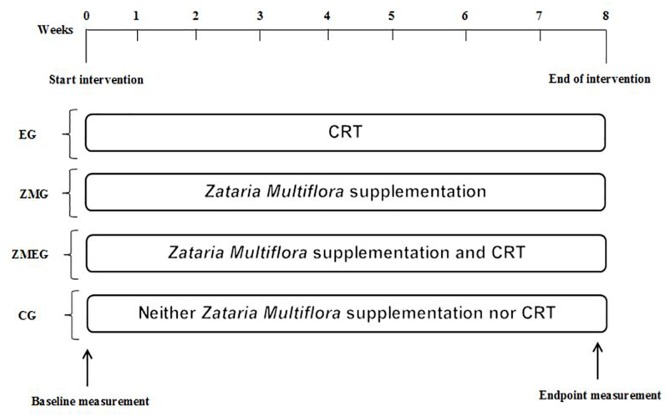
Experimental design of 8 weeks of CRT and *Zataria Multifloria* supplementation on adipokines response in postmenopausal. *CRT*, circuit exercise training; *EG*, exercise group; *ZMG*, *Zataria Multifloria* group; ZMEG, *Zataria Multifloria* and exercise group*; CG*, control group.

The experimental design of the present study is illustrated in Figure 1. This study included 48 postmenopausal participants. Participants were matched in terms of age, body weight, and body mass index and then divided into four groups: exercise (EG, *n* = 12), Zataria Multiflora (ZMG, *n* = 12), exercise and Zataria Multiflora (EZMG, *n* = 12), and control (CG, *n* = 12). Inclusion criteria for participation were: (1) 6 months or more pass menopause, (2) no prescribed drugs consumption before exercise session and/or alcohol addiction, (3) no history of regular exercise for at least 6 months, (4) no history of renal, hepatic, cardiovascular disease, diabetes and/or any physical injury or problem as a physical activity obstacle. Postmenopausal status was confirmed by postmenopausal levels of serum estradiol (<120 pmol/l) and follicle-stimulating hormone (FSH > 30 IU/l) and through examination by a gynecologist. Participants in experimental groups either performed CRT or supplemented with Zataria Multiflora, or their combination, for 8 weeks. Blood samples were collected at pre- and post-intervention for measuring selected adipokines. It should be noted that although no side effects have been reported with the dosage used in the present study, all participants were informed for any potential adverse effects of consuming herbal supplements prior to the study (Ghanbari-Niaki et al., 2018; Tayebi et al., 2018, 2019). Moreover, throughout the study participants had free access to a physician whenever they needed it. Finally, prior to the study beginning, all participants were accessed for any health issues (e.g., high blood pressure), which precluded their participation in the study. Therefore, participants were healthy which minimized the magnitude of potential unanticipated side effects occurring. This study was conducted according to the ethical principles of the Declaration of Helsinki, with informed written consent forms were signed by all participants.

### Zataria Multiflora Collection, Extraction, and GCMS

Zataria Multiflora leaves were collected in March. The leaves were cleaned, dried in the shade for 10 days, dried in an oven for 48 h at a temperature of 32°C and then powdered. 50 g of powdered sample was extracted using the water distillation method in a Clevenger apparatus at the boiling point for 3 h. The extract was filtered, dried on anhydrous sodium sulfate, transferred into a glass container (closed the lid) and stored at 4°C. The essential oil yield was calculated as dry essential oil volume divided by the initial dry powder weight multiplied by 100. In this fashion the yield calculated was 3% (Saei-Dehkordi et al., 2010). Agilent Gas Chromatography/Mas Spectrophotometry (GC-MS, Agilent, USA GC 7890A, MS 5975C, Agilant, United States) was used in order to identify the most volatile components of the powder of Zataria Multiflora. The GC was equipped with a HP-5 column (30 m length × 0.25 mm i.d., film thickness 0.25 μm coupled with an Agilent 5975 mass spectrometer). The column temperature was programmed at 50°C as an initial temperature, holding for 5 min, with 3°C increases per minute to the temperature of 240°C, followed by a temperature enhancement of 15°C per minute up to 300°C, holding at the mentioned temperature for 3 min. Injector port temperature was 290°C and helium used as carrier gas at a flow rate of 1.5 ml/min (Saei-Dehkordi et al., 2010). Afterward, the compounds in Zataria Multiflora essential oil were identified using fragmentation pattern in the database of wiley7n.l and NIST08 and also using the retention time in the chromatography column (Saei-Dehkordi et al., 2010). The ratio of peak area for each component to the total area was subsequently calculated. These results are shown in Table 1.

**Table 1:** The main components of the Zataria Multiflora as measured by GC-MS analysis.

Components	The area under the Peak (%)	Retention time (min)
Thymol	35:09	26.8
Carvacrol	36:5	22.9
*p*-cymene	22:03	7.7
γ-terpinene	23:55	6.8
α-pinene	16:53	3.2
β-caryophyllene	41:36	3
Carvacrolmethyl ether	33:26	2.4
α-terpinene	21:12	2.2
Spathulenol	48:36	2
Linalool	25:59	1.8
β-myrcene	20:00	1.5
	Total	80.3

### Zataria Multiflora Supplementation

A pilot study had been conducted to determine the dosage to be used in the present study. Briefly, a total of 20 participants were supplemented with Zataria Multiflora using three previously recommended dosages (100, 200, and 500 mg) for 1 month, followed by antioxidant and insulin resistance measurements at the end of the supplementation. The findings indicated that 500 mg consumption was the most effective dosage to significantly increasing or decreasing antioxidant activity and insulin resistance, respectively. Additionally, it should be noted that previous studies have reported no major side effects of 500 mg dosage (Ghanbari-Niaki et al., 2018; Tayebi et al., 2018, 2019). Therefore, a total of 500 mg of dry Zataria Multiflora leaves powder was placed in capsules. The ZMG and EZMG groups received a capsule (500 mg) of Zataria Multiflora every day after breakfast with 100 ml of water. The EG and CG groups consumed placebo capsules (500 mg wheat flour) after breakfast with 100 ml of water for a total of 8 weeks. The participants consumed the capsules on a daily basis in the presence of investigators.

### Exercise Training Protocol

Participants were familiarized with the environment and CRT movements for 1 week and then 1 repetition maximum (1-RM) for each of the given exercises was determined. The 1-RM for each exercise movement was calculated using Brzezinski equation (Ghanbari-Niaki et al., 2015). Training sessions were delivered using CRT format with alternation between upper-body and lower-body movements as well as multi-joint movements at the beginning of the movements (Ghanbari-Niaki et al., 2015). The exercises included: (1) Squat, (2) Chest press, (3) Leg press, (4) Standing Military Press, (5) Knee extension, (6) Seated cable rowing, (7) Knee Curl, (8) Biceps curl, (9) standing calf raise, (10) Triceps press, (11) Back extension, and (12) Abdominal crunch. Participants in the EG and EZMG groups performed movements at 55% of 1-RM for 8 weeks (3 sessions per week). It has been suggested to achieve an optimal cardiovascular health and also to reduce the risk of cardiovascular diseases, individuals should perform exercise training, in this case resistance training, with 40–55% of 1RM (American College of Sports Medicine [ACSM], 2017). Hence, in the present study we chose 55% of 1RM since it has been reported that this intensity can result in a greater amount of fat oxidation (American College of Sports Medicine [ACSM], 2017). Each exercise session included a 5 min warm-up and then followed by the 12 prescribed exercises, with duration of approximately 30 s at each exercise station. The number of repetitions at each station was recorded for the participants. In each session, two sets (turns) of 12 exercises were carried out such that between each set, there was a 3 min active rest period (Ghanbari-Niaki et al., 2015).

### Blood Sampling and Adipokines Measurements

Blood samples were collected at pre- and post-intervention from an antecubital vein in sitting position. Participants were required to meet the following criteria for blood sampling: (1) no exercise other than the prescribed exercise regime of the study at least 72 h before blood sampling, and (2) No drink or consume specific beverage or food such as coffee, dark tea, banana, and cereal 24 h before blood sampling. Blood samples were placed into EDTA (plasma) and sterile (serum) tubes. All samples were centrifuged and then were stored at −70°C until analysis. Following adipokines were measured using commercially available assay kits: (1) plasma visfatin (Cat. No. V0523EK, enzyme-linked immunosorbent [ELISA] assay kit, AdipoGen, Seoul, Korea); (2) serum omentin-1 (Cat. No. APO-54N-034, sandwich ELISA, ELISA kit, Apotech Corp., Switzerland); (3) plasma vaspin (Cat. No. CSB-E09771h, ELISA kit, Cusabio Biotech, Wuhan, China); (4) plasma FGF-21 (Cat. No. RD191108200R, sandwich ELISA kit, Biovendor, Heidelberg, Germany); (5) plasma adiponectin (Cat. No. RD191023100, sandwich ELISA kit, Biovendor, Heidelberg, Germany); (6) plasma leptin (Cat. No. RD191001100, sandwich ELISA kit, Biovendor, Heidelberg, Germany); and (7) plasma ghrelin (Cat. No. 171B7104M, ELISA kit - Bio-Plex 200 System, Bio-Rad Laboratories, Gurgaon, India). The Elisa machine used in the current project was made in Biohit Company in Finland. It was programmed to CV = 7.59 in terms of qualitative control. Moreover, in order to boost the measurement accuracy; all the tests were double checked.

### Statistical Analysis

All data are expressed as mean ± SD. The distribution of each variable was examined with the Shapiro–Wilk normality test. The overall effect of exercise and Zataria Multiflora supplementation was determined on selected adipokines prior to and post 8 weeks CRT using a mixed-model repeated-measures analysis of variance (group × time) with Bonferroni *post hoc* test. Statistical significance was set at *p* < 0.05. All data analysis was carried out using Statistical Package for the Social Sciences (SPSS version 23 for Windows).

## Results

### Physical Characteristics

Baseline physical characteristics of the participants are shown in Table 2. All groups were similar in terms of age, height, body weight, BMI (*p* > 0.05).

**Table 2:** Anthropometric characteristics of study participants.

	CG	ZMG	EG	ZMEG
Age (years)	56 ± 5	54 ± 4	58. ± 5	54 ± 6
Height (cm)	156.7 ± 3.5	160.9 ± 4.1	158.7 ± 3.8	159.2 ± 4.7
Body weight (kg)	68.7 ± 13.3	66.4 ± 10.9	67.8 ± 13.01	70.1 ± 10.8
BMI (kg/m^2^)	28.2 ± 1.9	25.9 ± 2.5	27.2 ± 1.8	27.8 ± 2.0

*CG, Control Group; ZMG, Zataria Multiflora group; EG, Exercise group; ZMEG, Exercise and Zataria Multiflora; BMI, Body mass index.*

### Selected Adipokines Levels Prior to and Post 8 Weeks CR

#### Visfatin

**FIGURE 2:**
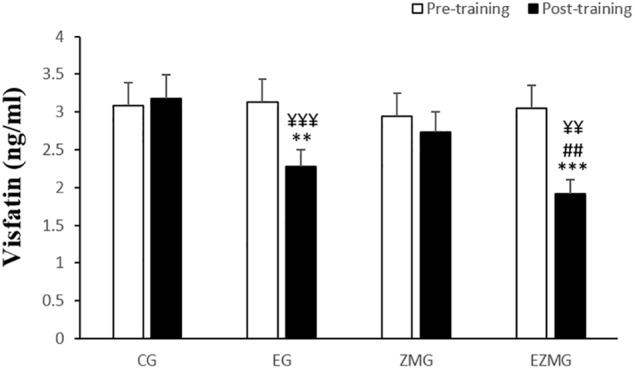
Visfatin levels in pre and post training. *CG*, control group; *EG*, exercise group; *ZMG*, Zataria Multiflora group; E*ZMG*, Exercise and Zataria Multiflora group. Data are expressed as means ± SD. ^∗∗^*P* < 0.01, ^∗∗∗^*P* < 0.001 versus post-test in CG group. ^##^*P* < 0.01 versus post-test in ZMG group. ^¥¥^*P* < 0.01, ^¥¥¥^*P* < 0.001 versus pre-test in same group.

There was a significant interaction between time and group for visfatin (Figure 2) [*F*(3,44) = 13.5, *p* = 0.000, η^2^ = 0.48]. Visfatin levels were significantly different between groups at post-intervention [*F*(3,44) = 12.5, *p* = 0.000, η^2^ = 0.46], with significant lower values in EG (mean = 2.27, *SD* = 0.49, *p* = 0.001) and ZMEG (mean = 1.91, *SD* = 0.58, *p* = 0.000) in comparison with CG (mean = 3.17, *SD* = 0.5). A significant lower value was also noted for ZMEG (mean = 1.91, *SD* = 0.58) in comparison with EG (mean = 2.27, *SD* = 0.49, *p* = 0.003) at post-intervention. There were significant differences over the time for visfatin in EG [*F*(1,44) = 30.4, *p* = 0.000, η^2^ = 0.4] and ZMEG [*F*(1,44) = 54.3, *p* = 0.000, η^2^ = 0.55], with lower values at post-intervention (EG = 2.27, *p* = 0.000, ZMEG = 1.91, *p* = 0.000) in comparison with baseline (EG = 3.12, *SD* = 0.6, ZMEG = 3.05, *SD* = 0.8).

#### Vaspin

**FIGURE 3:**
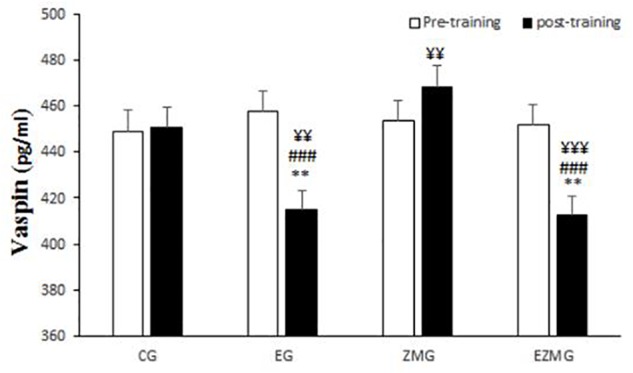
Vaspin levels in pre and post training. *CG*, control group; *EG*, exercise group; *ZMG*, Zataria Multiflora group; E*ZMG*, Exercise and Zataria Multiflora group. Data are expressed as means ± SD. ^∗∗^*P* < 0.01 versus post-test in CG group. ^###^*P* < 0.001 versus post-test in ZMG group. ^¥¥^*P* < 0.01, ^¥¥¥^*P* < 0.001 versus pre-test in same group.

There was a significant interaction between time and group for vaspin (Figure 3) [*F*(3,44) = 17.9, *p* = 0.000, η^2^ = 0.55]. Vaspin levels were significantly different between groups at post-intervention [*F*(3,44) = 13.2, *p* = 0.000, η^2^ = 0.47], with significant lower values in EG (mean = 414.7, *SD* = 7.5, *p* = 0.01) and ZMEG (mean = 412.4, *SD* = 7.5, *p* = 0.005) in comparison with CG (mean = 450.6, *SD* = 7.5). Vaspin levels were also lower at post-intervention in EG (mean = 414.7, *SD* = 7.5, *p* = 0.000) and ZMEG (mean = 412.4, *SD* = 7.5, *p* = 0.000) in comparison with ZMG (mean = 468.5, *SD* = 7.5), and in ZMEG (mean = 412.4, *SD* = 7.5) in comparison with EG (mean = 414.7, *SD* = 7.5). There were significant differences over the time for vaspin in EG [*F*(1,44) = 38.9, *p* = 0.000, η^2^ = 0.47], ZMG [*F*(1,44) = 4.7, *p* = 0.034, η^2^ = 0.98], and ZMEG [*F*(1,44) = 32.9, *p* = 0.000, η^2^ = 0.42], with lower values at post-intervention in EG (mean = 414.7, *SD* = 7.5, *p* = 0.000) and ZMEG (mean = 412.4, *SD* = 7.5, *p* = 0.000), and higher values in ZMG (mean = 468.5, *SD* = 7.5, *p* = 0.34), all compared to baseline (EG = 457.6, *SD* = 35.7, ZMEG = 451.8, *SD* = 31.7, ZMG = 453.5, *SD* = 35.1).

#### Omentin-1

**FIGURE 4:**
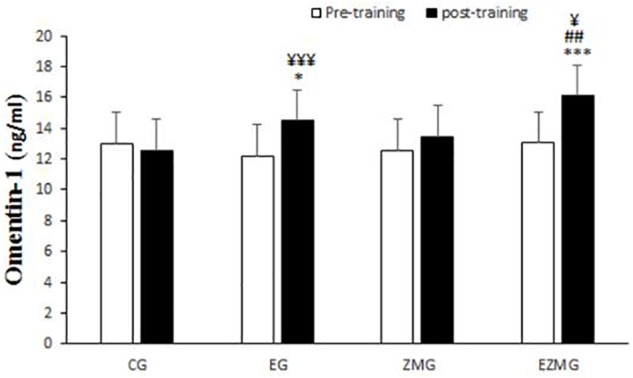
Omentin-1 levels in pre and post training. *CG*, control group; *EG*, exercise group; *ZMG*, Zataria Multiflora group; E*ZMG*, Exercise and Zataria Multiflora group. Data are expressed as means ± SD. ^∗^*P* < 0.05, ^∗∗∗^*P* < 0.01 versus post-test in CG group. ^##^*P* < 0.01 versus post-test in ZMG group. ^¥^*P* < 0.05, ^¥¥¥^*P* < 0.001 versus pre-test in same group.

There was a significant interaction between time and group for omentin-1 (Figure 4) [*F*(3,44) = 5.14, *p* = 0.004, η^2^ = 0.26]. Omentin-1 levels were significantly different between groups at post-intervention [*F*(3,44) = 9.2, *p* = 0.000, η^2^ = 0.38], with significant higher values in EG (mean = 14.5, *SD* = 0.49, *p* = 0.048) and ZMEG (mean = 16.1, *SD* = 0.49, *p* = 0.000) in comparison with CG (mean = 12.5, *SD* = 0.49). Omentin-1 levels were also higher in ZMEG (mean = 16.1, *SD* = 0.49, *p* = 0.003) in comparison ZMG (mean = 13.4, *SD* = 0.49). There were significant differences over the time for omentin-1 in EG [*F*(1,44) = 11.4, *p* = 0.002, η^2^ = 0.2] and ZMEG [*F*(1,44) = 19.8, *p* = 0.000, η^2^ = 0.31], with higher values at post-intervention in EG (mean = 14.5, *SD* = 0.49, *p* = 0.002) and ZMEG (mean = 16.1, *SD* = 0.49, *p* = 0.000) in comparison with baseline (EG = 12.2, *SD* = 1.8, ZMEG = 13.05, *SD* = 3.5).

#### Ghrelin

**FIGURE 5:**
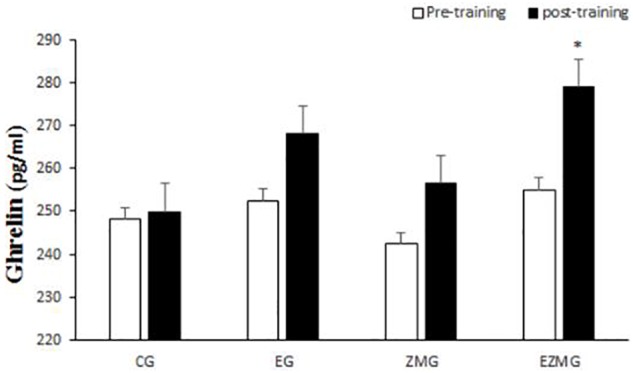
Ghrelin levels in pre and post training. *CG*, control group; *EG*, exercise group; *ZMG*, Zataria Multiflora group; E*ZMG*, Exercise and Zataria Multiflora group. Data are expressed as means ± SD. ^∗^*P* < 0.05versus post-test in CG group.

No significant interaction [*F*(3,44) = 0.8, *p* = 0.46, η^2^ = 0.05] and group [*F*(3,44) = 0.7, *p* = 0.54, η^2^ = 0.4] effects were noted for ghrelin (Figure 5). However, a significant main effect for time [*F*(1,44) = 8.1, *p* = 0.001, η^2^ = 0.15] was noted for ghrelin, with higher values at post-intervention in comparison with baseline.

#### Adiponectin

**FIGURE 6:**
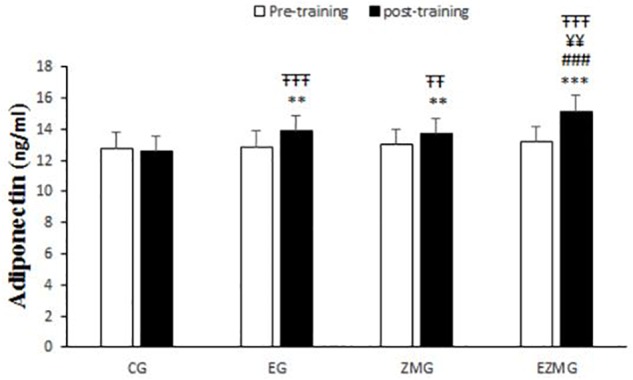
Adiponectin levels in pre and post training. *CG*, control group; *EG*, exercise group; *ZMG*, Zataria Multiflora group; E*ZMG*, Exercise and Zataria Multiflora group. Data are expressed as means ± SD.^∗∗^*P* < 0.01, ^∗∗∗^*P* < 0.001 versus post-test in CG group. ^###^*P* < 0.001 versus post-test in ZMG group. ^¥¥^*P* < 0.01 versus post-test in EG group. ^TT^*P* < 0.01, ^TTT^*P* < 0.001 versus pre-test in same group.

There was a significant interaction between time and group for adiponectin (Figure 6) [*F*(3,44) = 21.4, *p* = 0.000, η^2^ = 0.59]. Adiponectin levels were significantly different between groups at post-intervention [*F*(3,44) = 20.5, *p* = 0.000, η^2^ = 0.58], with significant higher values in EG (mean = 13.8, *SD* = 0.23, *p* = 0.002), ZMG (mean = 13.7, *SD* = 0.23, *p* = 0.006), and ZMEG (mean = 15.1, *SD* = 0.23, *p* = 0.000) in comparison with CG (mean = 12.5, *SD* = 0.23). Adiponectin levels were also higher at post-intervention in ZMEG (mean = 15.1, *SD* = 0.23) in comparison with ZMG (mean = 13.7, *SD* = 0.23, *p* = 0.001) and EG (mean = 13.8, *SD* = 0.23, *p* = 0.002). There were significant differences over the time for adiponectin in EG [*F*(1,44) = 26.2, *p* = 0.000, η^2^ = 0.37], ZMG [*F*(1,44) = 13.9, *p* = 0.001, η^2^ = 0.23], and ZMEG [*F*(1,44) = 102.6, *p* = 0.000, η^2^ = 0.7], with higher values at post-intervention in EG (mean = 13.8, *SD* = 0.23, *p* = 0.000), ZMG (mean = 13.7, *SD* = 0.23, *p* = 0.001), and ZMEG (mean = 15.1, *SD* = 0.23, *p* = 0.000) in comparison to baseline (EG = 12.8, *SD* = 1, ZMEG = 13.1, *SD* = 0.6, ZMG = 13.01, *SD* = 0.7).

#### Leptin

**FIGURE 7:**
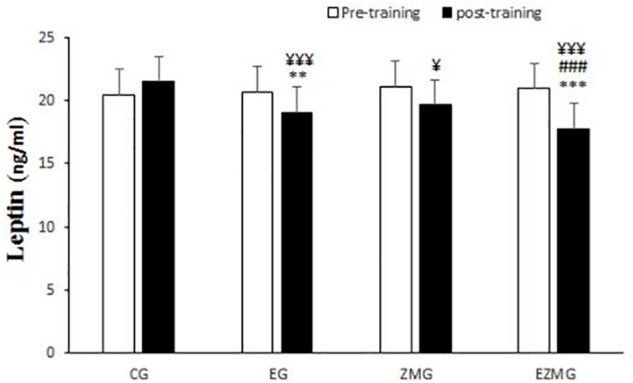
Leptin levels in pre and post training. *CG*, control group; *EG*, exercise group; *ZMG*, Zataria Multiflora group; E*ZMG*, Exercise and Zataria Multiflora group. Data are expressed as means ± SD.^∗∗^*P* < 0.01, ^∗∗∗^*P* < 0.001 versus post-test in CG group. ^###^*P* < 0.001 versus post-test in ZMG group. ^¥^*P* < 0.05, ^¥¥¥^*P* < 0.001 versus pre-test in same group.

There was a significant interaction between time and group for leptin (Figure 7) [*F*(3,44) = 25.9, *p* = 0.000, η^2^ = 0.63]. Leptin levels were significantly different between groups at post-intervention [*F*(3,44) = 10.1, *p* = 0.000, η^2^ = 0.4], with significant lower values in EG (mean = 19, *SD* = 0.49, *p* = 0.006) and ZMEG (mean = 17.7, *SD* = 0.49, *p* = 0.000) in comparison with CG (mean = 21.4, *SD* = 0.49). Leptin levels were also lower at post-intervention in ZMEG (mean = 17.7, *SD* = 0.49, *p* = 0.04) in comparison with ZMG (mean = 19.6, *SD* = 0.49). There were significant differences over the time for leptin in EG [*F*(1,44) = 23.3, *p* = 0.000, η^2^ = 0.34], ZMG [*F*(1,44) = 17.4, *p* = 0.000, η^2^ = 0.28], ZMEG [*F*(1,44) = 88.6, *p* = 0.000, η^2^ = 0.66], and CG [*F*(1.44) = 9, *p* = 004, η^2^ = 0.17]. with lower values at post-intervention in EG (mean = 13.8, *SD* = 0.23, *p* = 0.000), ZMG (mean = 13.7, *SD* = 0.23, *p* = 0.001), and ZMEG (mean = 15.1, *SD* = 0.23, *p* = 0.000), and higher values in CG (mean = 21.4, *SD* = 0.4), all compared to baseline (EG = 20.6, *SD* = 0.4, ZMEG = 20.9, *SD* = 0.4, ZMG = 21, *SD* = 0.4, CG = 20.4, *SD* = 0.4).

#### FGF21

**FIGURE 8:**
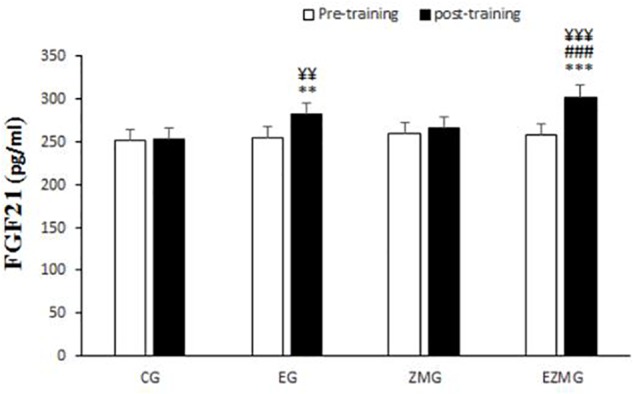
FGF21 levels in pre and post training. *CG*, control group; *EG*, exercise group; *ZMG*, Zataria Multiflora group; E*ZMG*, Exercise and Zataria Multiflora group. Data are expressed as means ± SD.^∗∗^*P* < 0.01, ^∗∗∗^*P* < 0.001 versus post-test in CG group. ^###^*P* < 0.001 versus post-test in ZMG group. ^¥¥^*P* < 0.01, ^¥¥¥^*P* < 0.001 versus pre-test in same group.

There was a significant interaction between time and group for FGF21 (Figure 8) [*F*(3,44) = 6.1, *p* = 0.001, η^2^ = 0.29]. FGF21levels were significantly different between groups at post-intervention [*F*(3,44) = 14.1, *p* = 0.000, η^2^ = 0.49], with significant higher values in EG (mean = 281.6, *SD* = 5.5, *p* = 0.004) and ZMEG (mean = 300.9, *SD* = 5.5, *p* = 0.000) in comparison with CG (mean = 252.7, *SD* = 5.5). FGF21levels were also higher at post-intervention in ZMEG (mean = 300.9, *SD* = 5.5, *p* = 0.000) in comparison with ZMG (mean = 265.7, *SD* = 5.5). There were significant differences over the time for FGF21in EG [*F*(1,44) = 12.5, *p* = 0.001, η^2^ = 0.22] and ZMEG [*F*(1,44) = 30.7, *p* = 0.000, η^2^ = 0.41], with higher values at post-intervention in EG (mean = 281.6, *SD* = 5.5, *p* = 0.001) and ZMEG (mean = 300.9, *SD* = 5.5, *p* = 0.000) in comparison with baseline (EG = 254.2, *SD* = 5.6, ZMEG = 258.1, *SD* = 5.6).

## Discussion

The beneficial effects of exercise-induced changes on select circulating adipokines in diverse populations have been well investigated (Sakurai et al., 2013). However, there does not appear to have been any study to date that has examined the combined effect of exercise training (i.e., resistance [CRT]) and herbal supplements (i.e., Zataria Multiflora) on circulating adipokines in postmenopausal women. To the best of our knowledge, this is the first study to report on the independent and combined effects of CRT and Zataria Multiflora on selected circulating adipokines in postmenopausal women. The present study tested the hypothesis that 8 weeks Zataria Multiflora supplementation along with CRT would have additional positive effects more than alone on circulating adipokines within postmenopausal women. The intervention was well controlled and the participants reported no side effects (e.g., nausea, weariness, headache, dizziness, and stomach problems or sleeplessness, etc.) during and/or following 8 weeks Zataria Multiflora supplementation. Our main findings in the present study were that the CRT and CRT plus Zataria Multiflora supplementation had more individual effects on most adipokines. However, Zataria Multiflora supplementation has little effect alone, indicating CRT as a critical element in the interventions we implemented.

### Visfatin

While plenty of studies have been conducted on visfatin and its role in metabolism, most of them suggest that visfatin is a highly expressed adipokine in visceral adipose tissue (VAT), which mediates glucose homeostasis (Seo et al., 2011). Moreover, it makes a nutrient-sensing mechanism, modulate B-cell function and finally prevent diabetes (Haus et al., 2009). Our results showed that 8 weeks of CRT was the single greatest reason for the significant decrease of visfatin plasma levels in postmenopausal women. Zataria Multiflora did not show an effect on visfatin without being combined with CRT exercise. Despite antioxidant and anti-inflammatory effect of Zataria Multiflora (Boskabady and Gholami Mhtaj, 2014) significant effect on visfatin in postmenopausal women was not observed. This study was the first study about the effect of Zataria Multiflora supplementation on visfatin. Therefore, it seems since visfatin is highly expressed in adipose tissue (Seo et al., 2011) and in the postmenopausal stage body fat usually increases (Goodpaster et al., 2005), the Zataria Multiflora could not modulate visfatin level by itself during or the duration of the study (8 weeks period) was not sufficient. On the other hand, a combination of supplementation and CRT was more effective than exercise alone.

### Vaspin and Omentin-1

Among the known adipokines, vaspin and omentin-1 play a key role in metabolism. In fact, there is a specific relationship among the body mass index (BMI), insulin sensitivity and glucose tolerance with the expression of vaspin *in vivo* (Heiker, 2014). Omentin-1 has an undeniable role in the enhancement of insulin-stimulated glucose uptake (Tan et al., 2008) or triggering Akt signaling in the lack or presence of insulin (Schäffler et al., 2005). Results from this study showed, as visfatin, CRT is the main reason for the change in the level of vaspin as well as omentin-1 plasma after 8 weeks period. In addition, Zataria Multiflora could increase vaspin plasma levels significantly without exercise. Thymol and carvacrol are the most important ingredients of Zataria Multiflora, which would bring some advantages in phase 1 metabolism, fatty acid oxidation and immune response (Hashemipour et al., 2013). It seems that the defined dosage of Zataria Multiflora used in the present study can modify vaspin plasma level regardless of CRT and can be a suitable supplement to decrease inflammation associated with this biomarker. To that end, vaspin has been reported to inhibit NF-KB and the expression of TNF α- and interleukin-1 induced adhesion molecules and to protect vascular endothelial cells. Various studies have shown the effects of insulin resistance and anti-inflammatory properties of vaspin (Liu et al., 2014). It has also been suggested that vaspin can increase the capacity of insulin absorption and insulin sensitivity, and ultimately affect glucose metabolism by regulating the proteolysis cascades and the gene expression level of the metabolic signaling pathways in the target tissues of the insulin (Liu et al., 2018). Additionally, vaspin has anti-inflammatory effects through inhibiting the expression of pre-inflammatory cytokines such as leptin, resistin and TNF-α. Furthermore, vaspin can reduce the inflammatory responses of smooth muscle cells caused by TNF-α by inhibiting ROS/PKC/NF-KB signaling (Liu et al., 2018). Zataria Multiflura likewise has potent antioxidants such as carvacrol and thymol that can reduce insulin resistance and inflammation by increasing vaspin levels. On the other hand, exercise training can reduce insulin resistance through GLUT-4 displacement, increased HDL and decreased triglyceride, LDL, and cholesterol, increased adiponectin and omentin 1 and reduced TNF-alpha reduced (Halverstadt et al., 2007; Jorge et al., 2011). In the present study, the administration of exercise training, independent of vaspin, and through these mechanisms just noted reduced insulin resistance and required no increase in vaspin.

### Gherlin, Leptin, and Adiponectin

The postmenopausal life stage is accompanied with the decline of estrogen levels in women, which leads to higher visceral fat percent (Mosca et al., 2007). Irregular hemostasis may make weight loss difficult even with implying some rigorous weight loss programs (Mosca et al., 2007). Adipose tissue is an important endocrine organ, which regulate different metabolic processes (Fève et al., 2016). Peptides including ghrelin, leptin and adiponectin play an important role in changing body composition, mediating hemostasis and inflammation (Blüher, 2014). In a closer view, ghrelin, known as a hunger signal, triggers meal initiation, and usually its level should be increased during weight loss (Kotidis et al., 2006). Also, there is evidence of its role in gastrointestinal motility, glucose metabolism and anti-inflammation (Tack et al., 2006). On the other hand, leptin has the opposite effect of ghrelin; i.e., its levels fall during periods of starvation (Hellström et al., 2004), and it has a positive correlation with systematic inflammation or even atherosclerotic diseases (Myers et al., 2008). Adiponectin has a different pattern compared to two others and is not affected by food intake during a day (Small and Bloom, 2004). Its role in insulin sensation and setting up anti-inflammatory cascade has been completely proved (Brochu-Gaudreau et al., 2010). Dietary-induced weight loss is accompanied by the rise of ghrelin plasma concentration, while leptin and adiponectin, usually decreases and increases during weight loss period respectively (Kotidis et al., 2006). The results from this study show that the interaction of CRT and Zataria Multiflora supplementation is the most effective way to modulate ghrelin, leptin and adiponectin plasma levels in postmenopausal women. However, it seems, leptin and adiponectin were affected significantly by just Zataria Multiflora. Mpalaris et al. (2016) showed that there is an association between bone mineral content and those peptides, in a way that there is a positive correlation between leptin concentration and negative one among the adiponectin and ghrelin and bone mineral content of postmenopausal women. Tsaroucha et al. (2013) reported that all of these peptides singly could play a main role to mediate the pathogenesis of asthma in obese women. Therefore, according to these reports and the data from this study, it seems, Zataria Multiflora with its anti-inflammatory and metabolic effect could be a useful supplement for people who are subjected to the mentioned diseases, such as postmenopausal women. However, the interaction of CRT and Zataria Multiflora appears more efficacious.

### FGF21

It is a novel factor that has been shown to possess beneficial effects on lipid metabolism and insulin sensitivity in animal studies (Fève et al., 2016). Its cooperation with adipocytes leads to stimulate insulin-independent glucose uptake by protein expression of GLUT4, and it is a key target for transcription factor PPARa, which is involved in lipid metabolism (Fève et al., 2016). Overexpression of FGF21 in transgenic mice resulted in the resistance to diet-induced obesity and metabolic perturbation (Fève et al., 2016). More importantly, it has been shown, FGF21 has a role in modulation of lipid profile (Kharitonenkov et al., 2007), which probably is due to its role in activation of extracellular signal-regulated kinase 1/2 and Akt signaling pathways (Wente et al., 2006). With regard to our results, Zataria Multiflora did not have as substantial role a ad CRT to induce a rise FGF21 plasma levels in postmenopausal women, likely this is because of the deregulation of other hormones in these women, which lead to more resistance of lipid and glucose metabolism among them (Maggio et al., 2007). However, this point requires further researches. A limitation of the current study was that the strength gain was not assessed after 4 weeks training to adjust the exercise-training protocol. This may have helped to better explain the results.

In conclusion, our findings suggest CRT is a critical intervention element that can significantly modulate circulating levels of adipokines. Interestingly, these effects were intensified when Zataria Multiflora supplementation added to CRT regimen, whereas ZM alone had little effects on measured adipokines. This implies that the beneficial effects of CRT on circulating levels of adipokines could be amplified with simultaneous intake of ZM; that is, they act synergistically. Consequently, CRT alone or its combination with ZM could be considered by researchers and health practitioners as a non-pharmacologic means to modulate and/or minimize menopause-induced obesity through alleviating the increase risk of metabolic syndrome in this population. Hence, this could lead to improved postmenopausal women’s well being and health. It is also paramount to note that further studies are warranted to elucidate the potential specific mechanism(s) responsible for ZM supplementation and exercise training-induced alterations in circulating levels of adipokines.

## Ethics Statement

The whole study was approved by the Ethical Committee on Human Research (ECHR) of the University of Mazandaran (Iran) according to the declaration of Helsinki.

## Author Contributions

All the authors contributed to the conception and design of the study and to the data collection. AS, MA, AA-D, FM, AH, SS, GB, ABA, and HZ performed the data analysis and interpretation. AS, GJ, MA, FM, SS, GB, and HZ drafted the manuscript. GJ, AS, MA, AA-D, FM, AH, SS, GB, ABA, and HZ revised, read and approved the submitted version.

## Conflict of Interest Statement

The authors declare that the research was conducted in the absence of any commercial or financial relationships that could be construed as a potential conflict of interest.
